# Reports on the Hospitals of the United Kingdom

**Published:** 1909-10-30

**Authors:** Henry Burdett


### REPORTS
ON THE
### Hospitals of the United Kingdom.

By SIR HENRY BURDETT, K.C.B., K.C.V.O.

### QUEEN'S HOSPITAL, BIRMINGHAM.

This hospital was founded in 1840, incorporated in 1867, and has recently been so completely reconstructed and added to that, providing the oldest portions, in which the operation theatre is, were to be pulled down, as they readily might be, and replaced with modern up-to-date buildings in the nature of the recent extensions, this institution might be made one of the most attractive, well-thought-out and up-to-date hospitals in the country. The cost of scrapping the original hospital buildings referred to and their replacement as suggested would cost a relatively small sum, say from £15,000 to £20,000. It at present contains 132 beds. We hope there may be one or more citizens of Birmingham who will be glad to follow the example of King Edward, who has done so much for the hospitals. If such there be they will promptly provide the necessary funds for this urgent need, and so set an example which should perpetuate their name and hand it on to posterity with honour and benefit to those who come after.

### A Centre of Progress.

The foregoing suggestions are the result of an inspection which we made of this hospital on September 16, 1909. Every ward and room, we might almost say every brick in the old building, and much of the new, outside the recent additions, have been made familiar to the writer by years of association. Here he first began his work for the hospitals, here was born the uniform system of hospital accounts, and here most of the principles and plan which form the basis upon which the present system of training nurses rests were first put in action. Sands Cox claimed for the Queen's Hospital that its foundations were sure, and that its continuous development and efficiency, hygienically and otherwise, were guaranteed because its site rested upon a bed of old red sandstone. This robust faith of one of Birmingham's oldest and best-known characters is certainly justified to day, thanks largely to the admirable courage and ability which those at present responsible for its management have exhibited in the recent additions and reconstructions.

### Dr. Foxwell and his Work.

We hope that the memory of the late Dr. Foxwell, whose tragic death recently stirred the sympathy of the whole country, may find a suitable memorial within the walls of the Queen's Hospital, where his great intelligence and thoroughness are nobly represented by the plan and new units of construction incorporated in the splendid pavilion which has recently been opened for patients. This pavilion will bring home to the lay mind how closely the rapid advance in treatment, due largely to scientific developments in recent years, is identified with the best interests of the patients, their comfort and rapid recovery. The aseptic system, for example, it has been calculated, has added one million working days each year to the earning power of the artisans of London. What has this system done for the artisans of Birmingham, as represented by the reduction in the number of days each patient is now resident in the Birmingham hospitals? At the Queen's Hospital the average residence of each in-patient, which used formerly to exceed thirty days, fell in 1903 to 22 days and in 1908 was under 18 days. This represents a reduction by quite one-half of the period of residence which formerly prevailed, and on this basis 2,600 in-patients and their families and dependents benefited to the extent of at least 31,000 days' earning power during which these convalescent patients could be earning the fruits of their labour. It should be realised that not one of these 31,000 days would have been available for the purpose fifteen or twenty years ago, for most or all of them would have been spent in the hospital wards.

### The Artisans' Noble Example.

The artisans of Birmingham have set a noble example to the whole country by their liberal contributions to the hospitals. They provided the funds for the first great extension of the Queen's Hospital upwards of thirty years ago. We have, therefore, thought it a matter of great interest to them for us to point out that a visit to the new pavilion of the Queen's Hospital will enable them to realise something of the means by which science has produced the present lessened residence in hospitals. They will find rooms for students, both men and women, clinical rooms where necessary tests, inquiries and experiments are made by which the precise nature of diseases are ascertained and recorded. They will see a roof ward which, as it stands to-day, constitutes probably, though small, the most perfect unit of construction of its type to be found in the whole country. They will realise, especially if they visit afterwards the old, narrow and imperfect wards in the original hospital, what an abundance of light and air and space and brightness means to a patient when confined to a hospital bed. These spacious corridors, and the carefully selected material for floors, doors and fittings throughout the newer portions of this hospital, must cheer the hearts of the working men who see them, and make them realise, as every other citizen of Birmingham should realise, the privilege of lending a hand to aid the Queen's Hospital Committee to provide the maximum of efficiency in every department.

A Great Heart and Opportunity.

Will not some man or men with great hearts and the mind to realise the glorious possibilities which a relatively few thousand pounds would reduce to practice, come forward and agree to defray the cost of rebuilding the two old wings and operation theatre? The name of anyone who will do this will go down to history not only as one of Birmingham's worthiest citizens, but as a benefactor to his fellows whose name will endure for generations to come!

New Out-patient and Casualty Department.

These are well thought out and excellent in many respects, but they contain one grave defect which the architect should have prevented and must now promptly rectify. The w.c.'s for men and women are most objectionable, and may soon constitute a danger to health if they are not promptly removed to a more suitable and isolated position. They are really offensive at present, and it is unaccountable how this danger and abomination could have been overlooked or passed. At present this nuisance spoils what would otherwise be excellent work.

A great deal of originality is to be seen in the plans of certain Nurses' Homes of recent date. Thus at the Queen's Hospital, where the comforts of the nurses have on the whole been met in the arrangements of the New Home, we found a spacious recreation hall where roller skating was carried on with much energy and enjoyment. At the Raphael Home, Guy's, there is a fine marble swimming bath of ample dimensions, and most nurses' relaxation rooms have polished floors in the present day, to enable dancing to be indulged in throughout the winter months. So health and good spirits are promoted and everything in a modern hospital is kept smartly alive and mostly at its best. What a change compared with 40 years ago! when the nurses at this hospital had to sleep in the basement with brick floors, and sore throats and diphtheria were frequently to be found!

### WOLVERHAMPTON EYE INFIRMARY.

This is a compact, well-organised special hospital, with 41 beds. It was built upwards of 20 years ago, *i.e.* in 1887, and so its construction leaves something to desire. The internal management in all departments is good, and reflects credit on the present Matron, Miss M. J. Connell, who was trained at the Royal Sheffield Hospital and is full of enthusiasm.

There is a resident house surgeon, and a staff of six nurses, of whom five are probationers. There is a very spacious out-patient department, containing every facility for doing the work thoroughly. It includes an inner hall for casualty patients, of which there should be a large number to justify the accommodation provided. The operation theatre is unusually large, and contains two beds. It was explained that these beds are needed for children after an operation, so that other children may be operated upon in the theatre whilst the nurse and Matron attend to children in the beds as well as to the child on the table. Only one of the beds, we were informed, is habitually used. The fact that the patients are children and that screens are used aggravates, in our judgment, this reversion to a policy dating back to the middle ages. Screens are out of place, and so are beds in an operation theatre, and should find no place there. Children should not be treated differently from adults in matters of this kind and it is certainly unjustifiable in the present day to retain patients who have been operated upon in a theatre whilst an operation on another patient is proceeding. The plea that the smallness of the nursing staff renders such a step necessary cannot be admitted, nor will it be urged by the managers of a modern, up-to-date hospital. We shall hope to hear that the theatre at the Wolverhampton Eye Infirmary will henceforward be confined to the purposes for which it was built.

This hospital is responsible, we understand, for the supply of the ophthalmic needs of Wolverhampton and the district. In 1908, 6,673 new patients were treated, of whom 670 were in-patients. The number of accidents dealt with was 2,697, which may account for the large casualty accommodation. The hospital is well supported, for although its expenditure in 1908 only amounted to £2,694 it received in legacies no less than £2,770.

**The Royal Devon and Exeter Hospital** will be reported on next week.

